# Pre-Prosthetic surgical alterations in maxillectomy to enhance the prosthetic prognoses as part of rehabilitation of oral cancer patient 

**DOI:** 10.4317/medoral.17482

**Published:** 2011-12-06

**Authors:** Hisham El Fattah, Ashraf Zaghloul, Enric Pedemonte, Tomas Escuin

**Affiliations:** 1 MD.Oro-Dental And Maxillofacial Department. National Cancer Institute, Cairo University, Egypt; 2 MD.Surgical Department. National Cancer Institute, Cairo University, Egypt; 3 PhD, DDS.Area of prosthodontics. University of Barcelona. Spain; 4 PhD, MD, DDS. Area of prosthodontics.University of Barcelona. Spain

## Abstract

Objectives: After maxillectomy, prosthetic restoration of the resulting defect is an essential step because it signals the beginning of patient’s rehabilitation. The obturator used to restore the defect should be comfortable, restore adequate speech, deglutition, mastication, and be cosmetically acceptable, success will depend on the size and location of the defect and the quantity and integrity of the remaining structures, in addition to pre-prosthetic surgical preparation of defect site. Preoperative cooperation between the oncologist surgeon and the maxillofacial surgeon may allow obturation of a resultant defect by preservation of the premaxilla or the tuberosity on the defect side and maintaining the alveolar bone or teeth adjacent to the defect. This study evaluates the importance of pre-prosthetic surgical alterations at the time maxillectomy on the enhancement of the prosthetic prognoses as part of the rehabilitation of oral cancer patient. Study Design: The study was carried out between 2003- 2008, on 66 cancer patients(41 male-25 female) age ranged from 33 to 72 years, at National Cancer Institute, Cairo University, whom underwent maxillectomy surgery to remove malignant tumor as a part of cancer treatment. Patients were divided in two groups. Group A: Resection of maxilla followed by preprosthetic surgical preparation. Twenty-four cancer patients (13 male – 11 female). Group B: Resection of maxilla without any preprosthetic surgical preparation. Forty-two cancer patients (28 male-14 female). Results: Outcome variables measured included facial contour and aesthetic results, speech understandability, ability to eat solid foods, oronasal separation, socializing outside the home, and return-to-work status. Flap success and donor site morbidity were also studied. Conclusions: To improve the prosthetic restoration of maxillary defect resulting maxillary resection as part treatment of maxillofacial tumor depends on the close cooperation between prosthodontist and surgeon, by combination of pre-prosthetic surgery during maxillectomy and prosthodontic technique.

** Key words:**Maxillectomy, pre-prosthetic surgery, quality of life, oral cancer.

## Introduction

In the past 25 years, Head and neck Surgery has subjected to rapid progression in surgical technique and technology that has led to respectability of larger and more extensive cancers tumors of the head and neck region and led to a larger number of patients with extensive post- surgical defects and subsequent physiological consequences that, without the field of maxillofacial rehabilitation, would lead to decreased patient satisfaction and poorer post-operative outcomes. Curing the cancer should not be allowed to obscure the importance of the quality of the patient’s life. Success depends upon both the judgment and skill of the therapist, and the post-treatment anatomic, physiologic, and psychological makeup of the patient. Treatment of the patient with cancers of the maxilla and hard palate is complex and results in significant functional and aesthetic sequelae (1,2). These may include collapse of cheek and infraorbital soft tissues, loss of hemi palate and oral phase of deglutition, difficulty with articulation and orbital complications (3).

This paper highlights the salient principles and discusses the different types of pre-prosthetic surgery after following maxillectomy that may be indicated for the management of maxillary defect resulting from maxillectomy as part of cancer treatment.

Now a day the debate about prosthetic obturation and surgical reconstruction of maxillary defects has never stopped. Recently multiple surgical approaches have been advocated to address some of the problems associated with traditional reconstructive approaches. Free flaps including rectus abdominus, radial forearm, lateral arm, fibula, iliac crest, and scapula have been used to reconstruct the maxilla and defects following maxillectomy and in many centers are considered the standard of care for primary reconstruction (1). These approaches successfully obliterate the maxilla and most often the orbital spaces. They close the palatal defect and can provide repair to the facial skin. This approach, however, is limited by difficulty in controlling certain aspects of facial contour, soft tissue prolapse, poor facial skin color match, and loss of direct tumor surveillance (4-7). Chandra et al. (8) and many researchers (9) reported the majority of maxillary defects can be ideally reconstructed with a simple obturator to restore oral functions and cosmetics following surgery because placement of larger obliterative flaps does not negatively impact on the ability to recognize tumor recurrence. Additionally surgical restoration of large defects is technically difficult and requires multiple procedures and hospitalizations, which may be further complicated if radiotherapy has been performed (10). To stabilize prosthetic restoration of large palatal defects, the surgeon must supplement bone to increase the area of the palatal arch. This can be achieved by the addition of vascularized or non-vascularized bone into which osseointegrated implants can be placed and a stable fulcrum line reestablished or by engaging tissue undercuts (11,12). Furthermore, restorations of abutment teeth used to retain an intraoral maxillofacial prosthesis must be sound and noncarious (1,13-15). The unfavorable forces are a particular problem in the edentulous patient or in previously irradiated patients whose teeth are absent or poorly suited to withstand the stresses of a clasp.

 As the maxillectomy patient requires maximal distribution of forces, the cheek will be an area of contact with the obturator. The thick squamous epithelium of a split-thickness skin graft will resist the wear and tear applied by the obturator. Genden et al. (16), illustrated that soft tissue flaps are effective for relining the oral cavity and separating the oral and nasal cavities. However, placement of a soft tissue flap obliterates the maxillectomy cavity and eliminates the retentive properties of the mucocutaneous scar band and the medial palatal shelf, thereby adversely affecting the prognosis for a stable tissue-borne dental prosthesis. Furthermore, the absence of bone will prevent the placement of osseointegrated implants.

Many authors describe the importance of preserving premaxillary segment by the fact that the residual premaxillary segment generally provides adequate volume and density of bone for the placement of implants (17,18). Alternative sites include posterior alveolar ridge, maxillary tuberosity, and the zygoma. The combination of zygomatic and standard endosseous implants have also been reported as an alternative option to reconstruct patients after extensive resection of the maxilla (19).

Removing the inferior turbinate, the prosthesis can be contoured to fit into the nasal cavity. This vertical height will resist the rotational forces applied during mastication. In addition, by adding the nasal cavity, a larger surface of bone will be utililized to distribute force during mastication.

The coronoid process removal can be considered as another surgical modification to prevent displacement of obturator or causing mucosal irritation. In resections that extend posterior into the soft palate it may be advisable to remove the coronoid process. Otherwise, as the mandible moves downward and forward the coronoid process may displace the distolateral aspect of the obturator resulting in mucosal irritation. Postoperative pain and limitation of mandibular movements is observed in cases wen coronoid process is preserved (not removed) (11).

If more than a small area of the floor of the orbit is resected, it should be repaired to prevent enophthalmos. Epiphoria is uncom-mon; when it occurs, it is related to scarring of the nasolacrimal duct or due to traumatic blockage of lymphatic drainage of the area (20).

## Material and Methods

This study was carried out on 66 patients whom were treated at National Cancer Institute, Cairo University between 2003-2008 whom under went immediate prosthetic reconstruction after maxillectomy surgery to remove malignant tumor as apart of cancer treatment.

 Patients were divided into groups according to preprosthetic surgical preparation before prosthetic restoration: Group A: Resection of maxilla followed by preprosthetic surgical preparation. Twenty-four cancer patients (13 male, 11 female). Group B: Resection of maxilla without any pre-prosthetic surgical preparation. Forty-two cancer patients (28 male, 14 female).

Patients with tumor extended to glob or skull base and who received radiotherapy before surgery were excluded from this study. Patients were followed up for period ranging 18-30 months.

Pre-surgical Procedure, for both groups:

Complete head and neck clinical exam was performed, with an assessment of overall facial symmetry and taking photographs for each patient to document changes and assess the response of the lesion to treatment. Ophthalmic examination was performed on the patients to check the range of extra-ocular motion, visual acuity, pupillary response, and signs of globe displacement. The nasal mucosal lining or fullness in the lateral or superior nasal cavity wall was also carefully assessed. Neck examination was performed to detect palpable lymph node metastases.

Pre-surgical dental and oral exam was done to determine number, location and integrity of the remaining teeth, the status of the dentition in the opposing arch and the size and arch form of the maxilla. Diagnostic casts of both arches were made. If time was available, the restoration of carious lesions, extraction of hopeless and prosthetically useless teeth and establishment of good periodontal status and oral hygiene procedures were done pre-prosthetically. Mandibular excursion was assessed for trismus and any possible sign of pterygoid musculature invasion.

General anesthesia with muscle relaxation was used for all types of maxillectomy. Either orotracheal or nasotracheal intubation were selected depending on the surgical approach. In some cases skin incisions were marked before the endotracheal tube is taped in place to avoid distortion of facial structures and skin lines. The patient was put in supine position in a 20° reverse. The eyes were protected carefully. Preoperative antibiotics were prescribed and continued until nasal packing was removed postoperatively.

**Figure 1:**
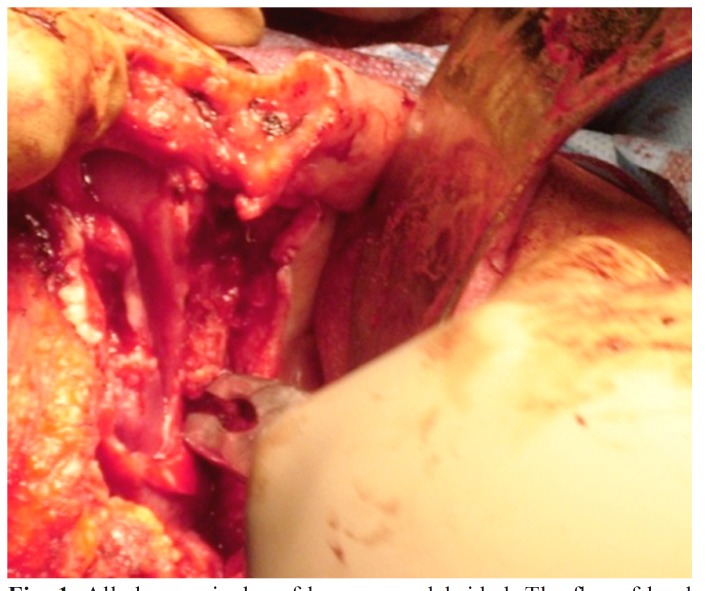
All sharp spicules of bone were debrided. The flap of hard palate mucosa is brought up over the cut bony edge of the palate. Exposed inferior turbinate is removed.

Surgical Approach, for both groups:

The choice of surgical approach was determined by the location, size, type, and aggressiveness of the tumor, the extent of the planned resection and by the preferences of the patient and the surgeon. Lesions usually were accessed via facial approach in conjunction with the transoral approach. If the lesion was located primarily anteriorly, maxillectomy were accomplished transorally without splitting of the lip. If there was any difficulty with exposure and resection or if the lesion was located laterally or far posteriorly, extraoral approach (Weber-Ferguson approach) was adopted to allow better access to the tumor. Complete exposure of maxilla was obtained through splitting the lip, extending the incision around the nose up to the orbit and along the eyelid. The mucosal incisions were outlined to give 5 to 10 mm margin around the tumor depending on the histopathology observed in the biopsy. These incisions were made through the periosteum. The periosteum was elevated to expose sufficient bone to permit cutting with osteotome and /or Giggly saw ( Fig. 1).

Standard Resection, for both groups:

The cut along infraorbital rim and superior anterior maxillary wall was made with a high-speed oscillating saw with a fine blade. The level at which this superior cut was made is determined by the extent of the resection. The line of transection was continued through the nasal process of the maxilla medially and downward through the piriform aperture. Laterally, the cut extends to the zygomatic process of the maxilla and around the posterolateral aspect of the sinus.

The line of transaction in the maxillary alveolus can run between two teeth if a suitable gap is evident, otherwise the tooth was extracted and the cut was made through the extraction site. Osteotome was used to cut horizontally through lateral nasal wall inferiorly at level of nasal floor (inferior meatus).

The hard palate was cut with a power saw. Once all the bone cuts are complete, an osteotome was used to connect them. Bleeding was controlled with large lap packs initially, then with bipolar cautery and figure-of-eight suture ligatures through pterygoid muscles. Bleeding from the internal maxillary artery was controlled by ligatures or legating clips.

A variable number of implants (in case of request) were inserted, after tumor surgery and before insertion of immediate surgical obturation, to be used later as anchorage for prosthesis.

Three obturators were delivered for the all over the period of treatment and rehabilitation. Obturators include three classes: surgical obturator (placed at the time of surgery); an interim surgical obturator (fabricated to aid in the healing of tissues during the recovery period 2-3 weeks after surgery) and after three months, a definitive obturator.

All surgical specimens were sent for histopathological examination to assess the tumor free resection margins and also to confirm the preoperative histological diagnosis.

Alternative Surgical Procedures (Pre- prosthetic surgical procedure), only for group A.

**Figure 2:**
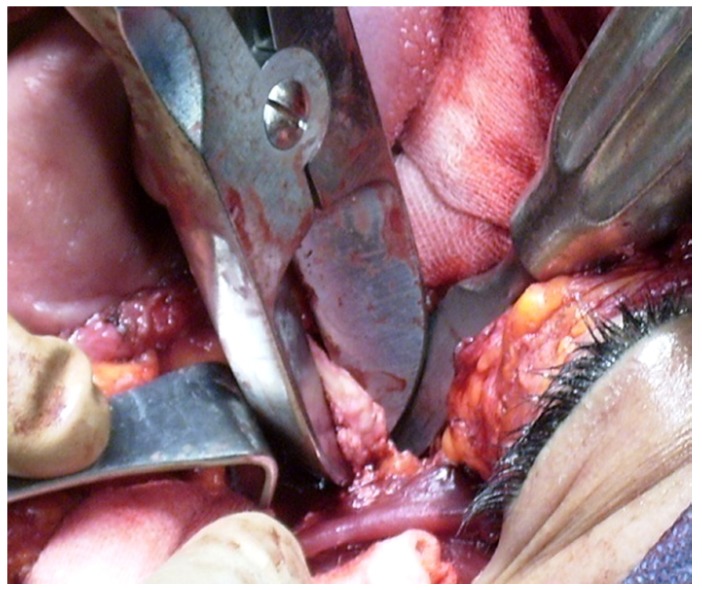
A split thickness skin graft was applied under the soft tissue flap to line the surgically produced cavity especially the check row area.

**Figure 3:**
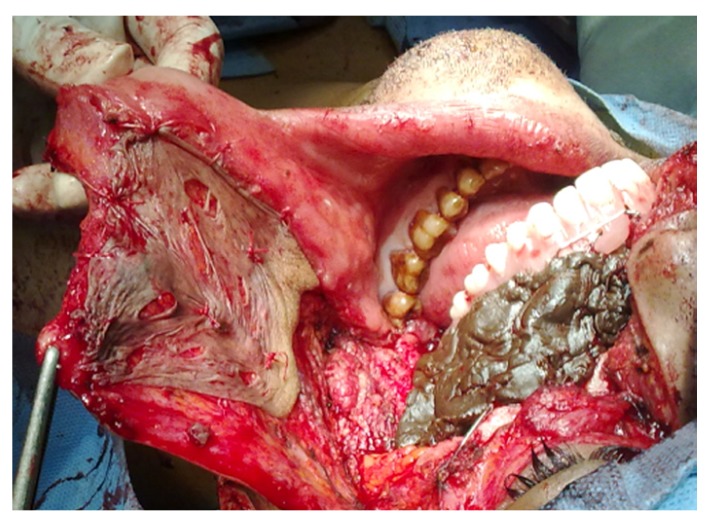
Coronoidectomy was performed.

• The horizontal incision of Weber Ferguson incision (from the lower eye led to the outer acanthus) was made 2mm close to the eye lash. An incision was made in the gingivobuccal sulcus and the mucosa of the hard palate maintaining adequate margin and using monopolar electrocautery. Incisions were made circumferentially through all the soft tissues up to the anterior wall of the maxilla and the hard palate. The infraorbital nerve was preserved if not affected with the disease process. An attempt was made to retain as much as possible of the hard palate consistent with adequate tumor free margin (Fig. 1).

• The cut of hard palate mucosa was made lateral to the planned cuts in the hard palate bone to create a mucosal flap, which was used to cover the cut bony edge of the hard palate, held in place with several Vicryl sutures. All sharp spicules of bone were debrided (Fig. 1).

• A split-thickness skin graft, 0.014 to 0.016 in. thick, was harvested from the anterolateral thigh and used to reline the raw buccal mucosa area. The graft was sutured to the cut edge of the buccal mucosa with 4-0 chromic catgut. Xeroform and strip gauze coated with antibiotic ointment were gently packed into the defect to secure the skin graft. The previously fabricated dental obturator was wired to the remaining teeth to hold the packing in place (Fig. 2).

• Removal of any exposed inferior turbinate, regardless of oncologic necessity. Prohibits the potential for subsequent inferior turbinate edema and descent into the oral cavity.

• If any teeth may be extracted to allow bone cuts through tooth sockets, adjacent teeth were preserved. If the mucosa covering maxillary antrum was not diseased, it was not removed.

• The premaxillary anatomy was preserved if the preservation did not compromise the oncologic objective to provide greater prosthetic support and stability.

• Coronoidectomy was performed in the operated side (Fig. 3).

Obturation of surgical defect, for both groups:

The fabricated surgical obturator was inserted immediately after removal of the neoplasm and fitted in place followed by modeling of gutta percha after repositioning of the cheek flap to regain normal contour of the face. Tension on the check flap was avoided. Then gauze pack was placed with its end emerging through the nose to facilitate its removal and allow irrigation through the nose during the period of initial healing. The obturator was ligated with 18-gauge wire to the remaining teeth and the zygomatic process to provide a stable prosthesis . In dentulous patients, a combination of stainless steel wire clasps, interdental wires or transalveolar ligation was used to stabilize the obturator. In the edentulous patient and after bilateral maxillectomy, stabilization was attained with transalveolar ligature wire and/ or bilateral zygomatic arch or supraorbital rim suspensors wires.

Postoperative Care, for both groups:

A nasogastric tube was placed at the end of the surgery. Most of the patients were able to begin a liquid diet and advance to a soft diet within a few days after operation. A soft diet was being continued for at least 2 weeks. Oral rinses and flushes with normal saline or half-strength hydrogen peroxide were being performed at least four times daily and after meals. Facial incisions were cleaned twice daily and coated with antibiotic ointment. Facial sutures are removed 5 to 7 days after operation.

The obturator and the packing were removed from the cavity in 10 to 14 days by cutting and removing ligature wires with wire cutter and the wound was irrigated with saline. Interim obturator was placed to keep oral competence. A final obturator was placed when healing was completed and the cavity stabilized, supported by implants if they were inserted.

All patients were instructed for jaw muscle training through physiotherapy to regain normal mouth opening, improve chewing efficiency and speech sound.

Outcome Measures:

Hospital stay or days spent postoperatively was recorded for each patient.

Facial contour and aesthetic results were assessed through postoperative photographs using a modified scale originally described by Funk et al. (21). Patients were assigned a numerical score to; 1: no deformity (an operative side that resembled the appearance of the non operative side in contour and symmetry with no ectropion or enophthalmos); 2: minimal deformity (included only minor soft tissue and skeletal asymmetry with minor ectropion or enophthalmos); 3: moderate deformity(involved a closed orbit without a nasocutaneous fistula, no exposed prosthesis, moderate ectropion or enophthalmos, and moderate soft tissue asymmetry or skeletal deformity as compared with the non-operative side); 4: severe deformity (consisted of gross soft tissue asymmetry, gross skeletal deformation, nasocutaneous fistula, exposed prosthesis, or severe ectropion or enophthalmos).

Speech understandability scale was modified from List et al. (22) 1: understandable; 2: understandable most of the time with occasional repetition necessary; 3: usually understandable but face-to-face contact necessary; 4: difficult to understand; 5: never understandable with written communication necessary.

Ability to eat solid foods was scored using the following criteria; 1: full range of solids with no restrictions; 2: minimally restricted solids with few specific exclusions (e.g., bread crumbs); 3: variety of solids taken but facilitated by increased moisture or liquid chasers; 4: minced, moist, or soft diet; 5: pureed solids; 6: no solids.

 Oronasal separation was scored as follows; 1: no evidence of velopharyngeal incompetence or nasopharyngeal reflux or nasal regurgitation of liquids; 2: mild inconsistent nasal emission, nares constriction, hypernasality, or nasal regurgitation or reflux; 3: moderate and consistent nasal emission, nares constriction, inappropriate nasality, or reflux or regurgitation; 4: severe or frequent nasal emission, nares constriction, and inappropriate nasality; 5: constant and continuous nasal emission, nares constriction, hyper nasal resonance.

Socialization also was noted, if the patient socialized outside the home; 1: frequently; 2: occasionally; 3: social event only; 4: only in case of emergency; 5: disabled.

Statistical analysis: Statistical Package for Scientific Studies SPSS version for Windows (SPSS Inc, Chicago, Ill) was used for analysis. Frequencies and basic descriptive statistics were conducted including means, standard error of the means, and ranges to illustrate characteristics of the patients and the postoperative. Student’s t-test, Mann-Whitney test and Pearson’s Chi square were performed to examine differences in outcome measures between patients with and without preprosthetic surgical intervention.

## Results

The main complications which were observed after maxillectomy surgery were ranged from case to case as follows; 1: Enophthalmos and hypophthalmos, which create a cosmetic deformity. 2: Infraorbital nerve injury, which results in anesthesia or paraesthesia of the ipsilateral cheek and upper lip. On occasion, the infraorbital nerve may have to be sacrificed as part of the planned resection. 3: Epiphoria, caused by scarring of the nasolacrimal duct. 4: Difficult retention of the dental prosthesis, prevented by careful preoperative evaluation and choice of reconstructive method. In select cases, free tissue reconstruction without a dental prosthesis may be optimal.

Hospital stay: Frequencies and basic of data on sex, age, hospital stay, contour deformity, follow-up, survival, speech, diet, and oronasal separation were calculated, including means, standard error of the means, and ranges and were tabulated to summarize the outcome score. Patients in Group B had significantly longer hospital stays (10.26 ±3days) when compared with patients in Group A (7.5 ±2.09 days) (P = 0.001). Group B patients reported better eating solid foods, speech understandability and oronasal separation when compared with Group A (p< 0.001). However, both facial contour and socialization scores were borderline statistically significant where Group A patients had lower scores denoting better outcome ( Table 1 and  Table 2).

**Table 1:**
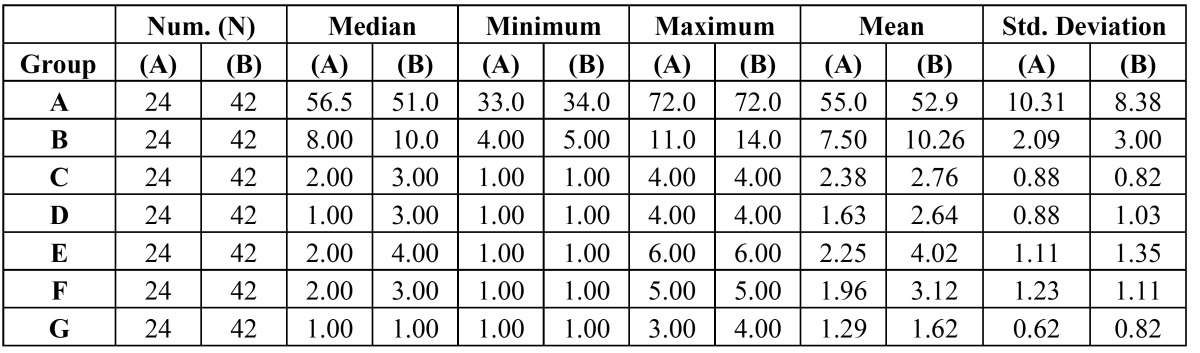
Frequencies and basic Data on age (A), hospital stay (B), facial contour deformity (C), speech understandability (D), ability to eat solids (E), oronasal separation (F) and socialization (G).

**Table 2:**
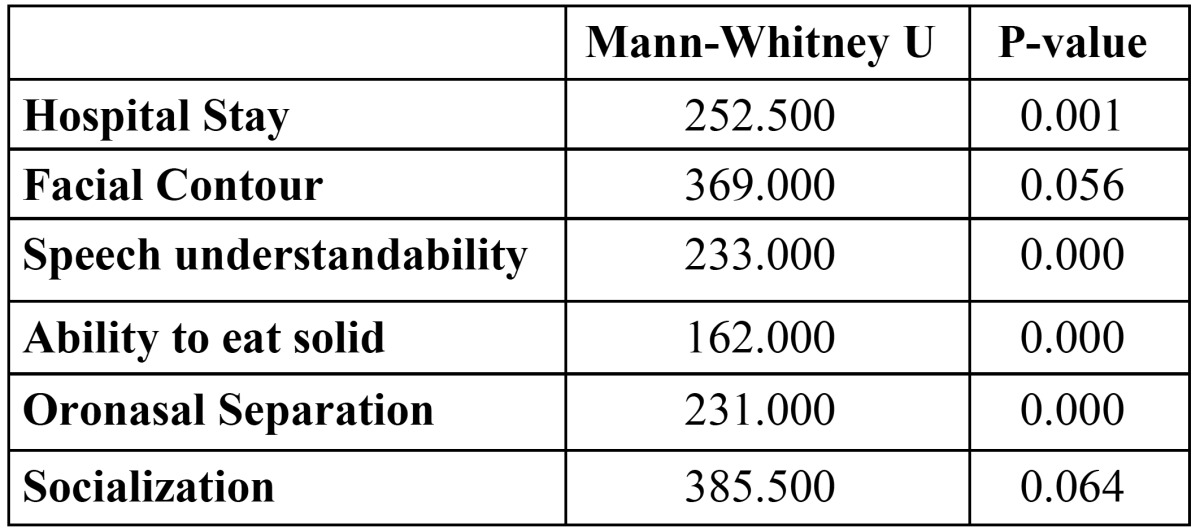
Comparison between 2 groups (test Mann-Whitney U (U).

Facial contour and aesthetic results: The mean ± SEM facial Contour score ( Table 1) of this patient population was 2.38=+0.88 for Group A and 2.76=+0.82 for Group B. Subgroup analysis demonstrated that there is no significant impact facial contour scores, 12.5 % in Group A and 7.1% in Group B (P<0.001). Moderate deformity involved a closed orbit without nasocutaneous fistula, no exposed plating, moderate ectropion or enophthalmos, and moderate soft tissue asymmetry or skeletal deformity as compared with the nonoperative side was reported in 25 % of Group A and 50% of Group B . Severe deformity, 12.5 % in Group A and 16.7% in Group B consisted of gross soft tissue asymmetry, gross skeletal deformation, nasocutaneous fistula, exposed plating, or severe ectropion or enophthalmos ( Table 3).

**Table 3:**
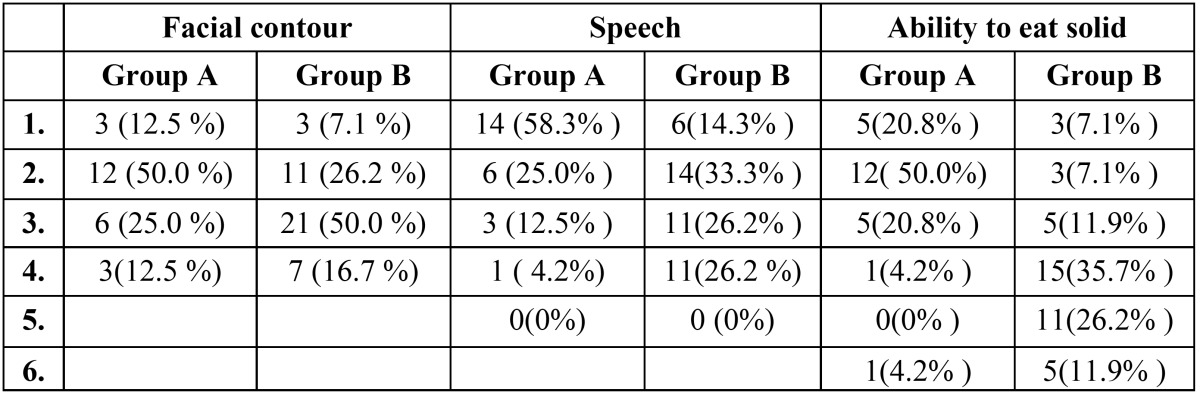
Facial contour and aesthetic results scale. The speech understandability scale. Ability to eat solid foods scale.

Speech understandability: Patients in Group B tended to have worse speech scores (2.64 ±1.03) than those in Group A (1.63± 0.88). This difference in speech score approached statistical significance (P <0.001). The speech understandability scale revealed 58.3% of Group A and 14.3% of Group B being understandable; 25% in Group A and 33.3% in Group B being understandable most of the time with occasional repetition necessary. More difficult to understand was reported in Group B (26.2%) when compared with Group A (4.2%) ( Table 3).

Ability to eat solid foods: The mean ± SEM diet score of patients in Group A (2.25 ± 1.11) demonstrated a trend toward significantly worse diet scores than those of patients in Group B ( 4.02 ± 1.35) (p<0.001) ( Table 1 and  Table 2). More patients in Group A were able to eat solid foods either with no restrictions (20.8%) or with few specific exclusions (50%) or facilitated by increased moisture or liquid (20.8%) compared with 7.1%, 7.1% and 11.9% in Group B respectively. On the other hand, more of Group B patients were eating either minced, moist, or soft diet (35.7%), pureed solids (26.2%), and 4.2% were not able to eat solids at all ( Table 3).

Oronasal separation: The mean score in Group A was significantly lower than in Group B ( Table 1). No evidence of velopharyngeal incompetence or nasopharyngeal reflux or nasal regurgitation of liquids was reported by 45% of Group A patients and only 9.5% in Group B. Mild inconsistent nasal emission, nares constriction, hyper nasality, or nasal regurgitation or reflux was reported by 33.3% of Group A patients compared with 16.7% in Group B. The 64.3% of patients in Group B experienced moderate or severe forms of consistent nasal emission, nares constriction, inappropriate nasality, or reflux or regurgitation ( Table 4).

**Table 4:**
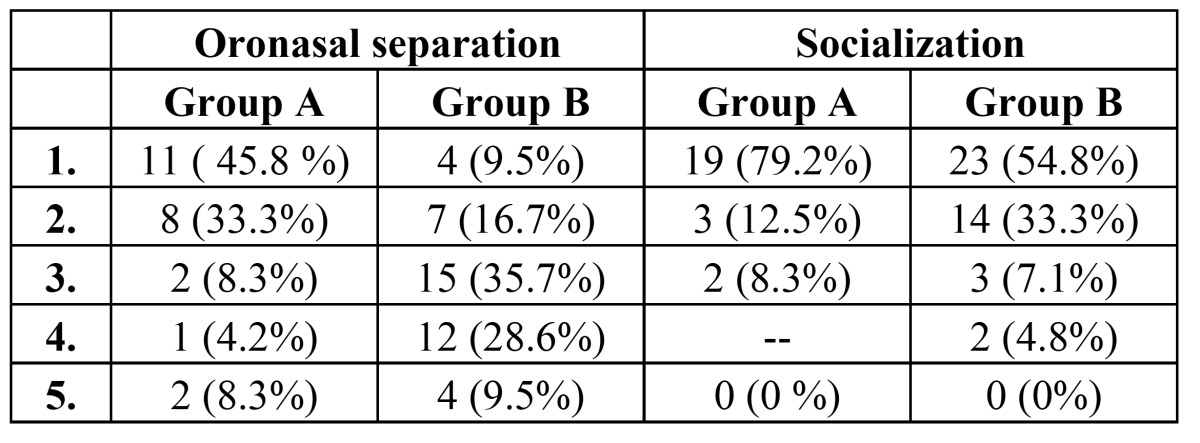
Oronasal separation scale. Socialization scale.

Levels of Socialization and return-to-work status: All patients in this study socialized outside the home either frequently or in emergency. Levels of socialization and return-to-work status were also studied in this population. All patients socialized outside the home either frequently (79.2% in Group A and 54. 8% in Group B) or occasionally (12.5% in Group A and 33.3% in Group B) or social event only (8.3% in Group A and 7,1% in Group B). All patients who were not disabled or retired at the time of reconstruction (100%) returned to work ( Table 4).

## Discussion

Although there are several factors influencing the prognosis of prosthetic reconstruction in patients whom had maxillectomy such as the size of the defect, availability of hard and soft tissues in the defect area to provide support for the prosthesis, proximity of vital structures, patient attitude, temperament, systemic conditions, and the patient’s ability to adapt to the prosthesis, this study adds the necessity to put in consideration the importance of preprosthetic surgical procedure at the time of maxillectomy.

In extra oral approach (Weber-Ferguson approach), the lateral incision should be continued laterally in the subciliary crease along the inferior eyelid or maximum 2 mm away from eyelid to the lateral canthus of the eye to avoid formation of preoccular edema due to interference with lymph drainage in this area which may cause facial disfigurement (2).

This study announces that preservation of all possible teeth and vigorous dental hygiene are important in the preoperative period to reduce problems in the postoperative period. The premaxillary anatomy should be preserved (if it does not compromise the oncologic objective) to provide greater prosthetic support and stability.

It is advised to placing the line of resection through the socket of an extracted tooth rather than attempting to cut between roots of adjacent teeth. Cuts between teeth sockets will result in loss of support for adjacent teeth and lead to loss of uninvolved teeth. Many authors recorded the importance of maintaining as much as possible of the hard palate in primary retention, support, and stability, especially ipsilateral palate preservation, which will allow a tripoding effect.

Removing the inferior turbinate, the prosthesis can be contoured to fit into the nasal cavity. This vertical height will resist the rotational forces and a larger surface of bone will be utililized to distribute force during mastication (23).

The flap of hard palate mucosa should be brought up over the cut bony edge of the palate and held in place with several sutures aiming to preserve bone in this area, accelerate healing during early healing phase and eliminates pain caused by pressure from the obturator on bar bone (2,4).

If the cheek flap is left to heal by secondary intention, the healing time will extend many weeks, and the area will be covered with respiratory epithelium from the nasal cavity and nasopharynx. This epithelium is problematic in two ways; 1: The tissue is easily abraded by future prosthesis, 2: This type of epithelium is secretary adding secretions that the patient must clean or have cleaned. Several publications (2,24,25) have devoted that retention of the obturator is aided by the band of scar tissue that forms at the junction of the mucosa and the skin graft.

Patients in Group B had significantly longer hospital stays compared with those patients in Group A this may be either due to acceleration of healing in Group A due to covering the check row area and palatal bar bone or due to early jaw function, which allow him to have his meal without the use of nasogastric tube. This, in addition to immediate prosthetic rehabilitation which correct facial disfigurement had positive psychological condition on the patient that allowed him to leave the hospital earlier.

The present study shows significant improvements in speech understandability, ability to eat solid food and oronasal separation. In similar study carried by Ducic and Oxford (10) approved that correctly constructed obturator will usually result in the return of normal speech and swallowing. Some researchers (24) focus on several surgical principles that should be utilized when performing lip-splitting incision to improve aesthetic results.

The speech understandability was improved in Group A compared with Group B, this may be due to restoration of functional anatomy of the maxillary sinus by well fit obturator. Literature reported that restoration of maxillary defect simulates the functional anatomy of the maxillary sinus and adds resonance to the speech (24-26).

Levels of socialization and return-to-work status was higher in Group A (79.2%) than in Group B (54, 8% ) these may be due the self confidence gained from the improvements in speech understandability, ability to eat solid food and complete oronasal separation due to retentive and stable prothesis which allowed the patient to freely communicate with other people.

The primary objective of rehabilitation is the restoration of appearance and function. How successfully this is accomplished, depends upon both the judgment and skill of the therapist, and the post-treatment anatomic, physiologic, and psychological makeup of the patient. In addition to assess and fabricate obturators for the patient, dental oncologist can also make suggestions to the surgeon for resections that will make the stability and comfort of the prosthesis better.

Treatment of the patient with cancers of the maxillary sinus and hard palate is complex and requires a multidisciplinary team approach at time of initial diagnosis and treatment planning. For maximal patient satisfaction and rehabilitation, the maxillofacial prosthodontist must have an active role in the pre and postoperative coordination of patient care and the head and neck surgeon must be aware of the assistance that the maxillofacial surgeon can offer in the treatment of this difficult and often devastating disease. The surgeon and the prosthodontist should be encouraged to work together to develop surgical and nonsurgical measures for achieving functional success of prostheses, success of surgical procedure, prevention of postoperative complications, and improved aesthetic and function results which can help with patient satisfaction. Surgery before prosthetic rehabilitation may be indicated to improve the existing anatomic configuration after ablative cancer surgery, reconstructive surgery, and/or radiation therapy. Multidisciplinary cancer care is required to achieve the best functional, physical, and psychologic outcomes.
